# A competitive formation of DNA:RNA hybrid G-quadruplex is responsible to the mitochondrial transcription termination at the DNA replication priming site

**DOI:** 10.1093/nar/gku931

**Published:** 2014-10-11

**Authors:** Ke-wei Zheng, Ren-yi Wu, Yi-de He, Shan Xiao, Jia-yu Zhang, Jia-quan Liu, Yu-hua Hao, Zheng Tan

Nucleic Acids Res. (2014) 42 (16): 10832–10844. doi: 10.1093/nar/gku764

The authors wish to apologize for errors in the last paragraph of the DISCUSSION section in this paper. The “RNA DQ” should read as “intramolecular RNA G-quadruplex (RQ)” at its first appearance and as “RQ” at the followings. The “DNA DQ” should all read as “DQ”.

With these corrections, the correct version appears below (starting from “Alternatively…”).

Alternatively, the RNA could also form an equivalent intramolecular RNA G-quadruplex (RQ) of three G-quartet layers by using the 13G-15G as the non-template DNA strand did in this work. However, the mutation in the 13G-15G in our work is expected to disrupt (M14/16) or promote (M3G) the formation of RQ, respectively, as to the DQ. But these showed little effect on the termination (Figure 8A, lanes 7 an 8 versus 5; Figure 8B, lanes 3 and 4 versus 1). This suggested that the RQ was largely irrelevant to the termination. On the other hand, because of the bulky T_3_ loop and bulge, the RQ might also be unstable as was the DQ. With the participation of G_3_ tracts from the DNA and RNA, the HQ of three G-quartets was much more stable and should be more competitive to form and to play a role in transcription termination.

